# Modified Thoracolumbar Interfascial Plane Versus Erector Spinae Plane Block for Postoperative Analgesia After Lumbar Discectomy: A Prospective Observational Comparative Study

**DOI:** 10.3390/jcm15062214

**Published:** 2026-03-14

**Authors:** Fatma Acil, Andaç Dedeoğlu, Okan Andıç, Meral Erdal Erbatur, Hülya Tosun Söner, Abdurrahman Çetin, Osman Uzundere, Cem Kıvılcım Kaçar, Erhan Gökçek

**Affiliations:** 1 Department of Anesthesiology and Reanimation, Diyarbakır SBÜ Gazi Yaşargil Training and Research Hospital, Diyarbakır 21070, Turkey; anderen77@hotmail.com (A.D.); okan_andic@hotmail.com (O.A.); hulyatosunsoner@hotmail.com (H.T.S.); osmanuzundere@gmail.com (O.U.); cem.kacar@hotmail.com (C.K.K.); gokcekerhan_44@hotmail.com (E.G.); 2 Department of Anesthesiology and Reanimation, Dicle University, Diyarbakır 21280, Turkey; merdalerbatur@gmail.com; 3 Department of Neurosurgery, Diyarbakır SBÜ Gazi Yaşargil Training and Research Hospital, Diyarbakır 21070, Turkey; acetin2147@gmail.com

**Keywords:** analgesia, laminectomy, postoperative pain

## Abstract

**Background:** Effective pain control after lumbar disc surgery is a key determinant of recovery. Therefore, we aimed to compare the effects of modified thoracolumbar interfascial plane block (M-TLIP) and erector spinae plane block (ESP) on postoperative pain control and opioid consumption. **Methods:** This prospective observational comparative cohort study included 96 patients aged 18–70 years with American Society of Anesthesiologists (ASA) physical status I–III who underwent elective single-level lumbar discectomy. Patients received either an M-TLIP block (Group M-TLIP, *n* = 49) or an ESP block (Group ESP, *n* = 47). Postoperative pain was assessed using visual analog scale (VAS) scores at 1, 2, 4, 8, and 24 h as the primary outcome. Secondary outcomes included opioid consumption, postoperative nausea and vomiting, Riker’s Agitation Sedation Scale (RSAS) scores, and patient satisfaction. Repeated pain measurements were analyzed using a linear mixed-effects model. **Results:** Postoperative pain scores were lower in the M-TLIP group compared with the ESP group, particularly during the early postoperative period. Linear mixed-effects modeling demonstrated a significant main effect of group and time, with the analgesic advantage of M-TLIP being most pronounced in the early postoperative hours and diminishing by 24 h. Total tramadol consumption within the first 24 h was significantly lower in the M-TLIP group (*p* = 0.039). Postoperative agitation, nausea and vomiting, and patient satisfaction scores were comparable between groups. **Conclusions:** These findings suggest that M-TLIP block may represent a clinically useful alternative to ESP block for postoperative analgesia in lumbar discectomy.

## 1. Introduction

Lumbar disc herniation is a common cause of severe back and leg pain, and persistent postoperative discomfort comparable to preoperative pain can impede early mobilization, necessitate higher analgesic doses, extend hospitalization, and decrease patient satisfaction [1].

Multidisciplinary perioperative strategies, known as enhanced recovery after surgery (ERAS) protocols, are now widely implemented across multiple surgical disciplines [2]. To achieve effective analgesia, methods such as NSAIDs, ketamine, opioids, lidocaine infusion, gabapentin, spinal-epidural injections, and interfascial plane blocks are increasingly utilized. Compared with neuraxial or plexus anesthesia, interfascial plane blocks offer effective analgesia with lower risk of neurological or motor complications. Common plane blocks in spine surgery include the erector spinae plane (ESP) block and the thoracolumbar interfascial plane (TLIP) block [3].

Forero introduced the erector spinae plane (ESP) block in 2016, while Hand [4] described the thoracolumbar interfascial plane (TLIP) block a year earlier, in 2015. Subsequently, Ahiskaloğlu et al. [5] developed a modified TLIP (M-TLIP) block with a lateral approach. This lateral technique aims to lower the risks of infection and neural injury by positioning the injection site at a greater distance from the operative field. However, studies on the efficacy of M-TLIP and ESP blocks in lumbar disk surgery are still ongoing.

In the context of lumbar discectomy procedures, the present study set out to determine whether M-TLIP block provides superior analgesia compared to ESP. Furthermore, secondary outcomes included data on postoperative sedation and agitation levels, nausea and vomiting, patient satisfaction, and the consumption of opioid analgesics both intraoperatively and postoperatively.

## 2. Materials and Methods

### 2.1. Study Design

This prospective observational comparative cohort study was conducted between October 2023 and February 2024 at the Health Sciences University Gazi Yaşargil Training and Research Hospital, after approval from the institutional ethics committee (Date/No: 03.03.2023/373).

Patients received either the modified thoracolumbar interfascial plane (M-TLIP) block or an erector spinae plane (ESP) block, and outcomes were prospectively collected for comparative analysis. No randomization was performed, and treatment allocation was based on routine clinical practice rather than a study-directed intervention.

The choice of block technique was determined by the single anesthesiologist routinely assigned to the neurosurgical operating room during the study period. Both techniques were part of standard clinical practice in this setting, and no predefined allocation protocol or randomization was used. Importantly, block selection was not based on patient demographic characteristics, ASA status, BMI, number of surgical levels, or anticipated pain severity. Patients were enrolled consecutively to minimize potential selection bias. The use of a single experienced anesthesiologist ensured technical consistency across procedures. Although selection bias cannot be completely eliminated in observational study designs, consecutive enrollment and standardized clinical decision-making were used to reduce allocation-related bias.

The study was conducted in accordance with the 2013 Declaration of Helsinki and adhered to the STROBE guidelines for observational studies (see Supplementary Material File S1: STROBE Checklist). This prospective observational comparative cohort study was registered at ClinicalTrials.gov (NCT05999253) prior to patient enrollment to enhance transparency regarding the study protocol and predefined outcomes. This registration does not indicate a randomized or interventional clinical trial design.

All outcome data were collected by clinicians who were not involved in the block procedures.

Detailed information regarding the surgical procedure, anesthesia management, and analgesia-focused block techniques was provided to all participants. Written informed consent was obtained from all patients prior to enrollment. The Visual Analog Scale (VAS) was explained to patients as a standardized tool for assessing postoperative pain.

### 2.2. Inclusion and Exclusion Criteria

A total of 112 patients were screened for eligibility. The inclusion criteria for the study were as follows: (1) patients aged between 18 and 70 years, (2) classified as ASA physical status I–III, (3) scheduled for elective lumbar discectomy for single-level or two-level lumbar disc herniation, and (4) able to provide written informed consent. Exclusion criteria: Patients were excluded if (1) they had more than two-level disc herniation, required multilevel (>2 level) laminectomy, (2) had infection at the block site, (3) coagulopathy, (4) morbid obesity (BMI > 35 kg/m^2^), (5) ASA > III, (6) allergy to local anesthetics, (7) chronic pain, (8) long-term opioid use, (9) psychiatric illness, (10) inability to provide consent, or (11) required emergency surgery.

### 2.3. Preoperative Period

A seasoned anesthesiologist conducted a thorough preoperative assessment in the ward for every patient. Age, gender, and BMI were the demographic variables collected. Each patient was taken to the operating room on the day of the surgery after they had fasted for 8 h before. Patient monitoring was performed in accordance with ASA standards, incorporating non-invasive blood pressure readings, pulse oximetry, and 2-lead electrocardiography.

A 20 gauge cannula was inserted into the antecubital region of the patients to establish intravenous access 30 min prior to surgery, after which they were transported to the preoperative block preparation room. Following premedication with intravenous midazolam (1 mg) and a low dose of fentanyl (50 μg), an anesthesiologist with more than five years of experience performed the plane block. Patients who developed a sensory block at the incision site, as confirmed by the hot–cold test, were enrolled in the study approximately 20–30 min after the block application, at which time general anesthesia was administered.

### 2.4. Pain Management

#### 2.4.1. M-TLIP Group

**Figure 1:**
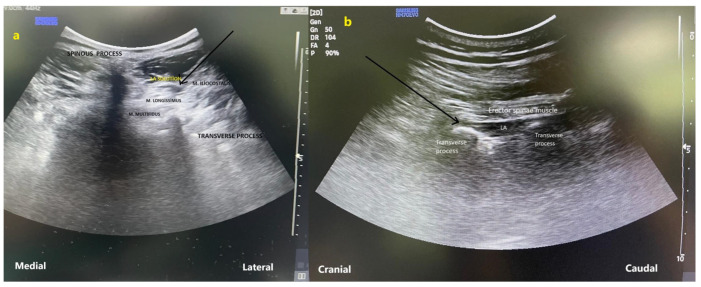
Ultrasound images demonstrating the anatomical landmarks and local anesthetic injection planes for the modified thoracolumbar interfascial plane (M-TLIP) block and the erector spinae plane (ESP) block. (**a**) Parasagittal ultrasound view showing the spinous process, transverse process, multifidus, longissimus, and iliocostalis muscles, with local anesthetic deposited between the multifidus and longissimus muscles (M-TLIP block). (**b**) Transverse ultrasound view illustrating the erector spinae muscle overlying the transverse process, with local anesthetic injected deep to the erector spinae muscle (ESP block). Arrows indicate the site of local anesthetic injection. M-TLIP: modified thoracolumbar interfascial plane; ESP: erector spinae plane.

In the preoperative block preparation room, the patients were positioned prone, and the site for block administration was sterilized. A low-frequency convex probe (Samsung HM70 EVO, Cheonho-daero, Gangdong-gu, Seoul, Republic of Korea) was sterilized and positioned vertically at the vertebral level designated for discectomy. Following the visualization of the spinous process, the probe was laterally adjusted to observe the multifidus, longissimus, and iliocostal muscles. A SonoPlex^®^ needle (Pajunk GmbH, Geisingen, Germany), measuring 80–100 mm and 20-gauge, was inserted in-plane into the interfascial plane located between the longissimus and iliocostal muscles (Figure 1). After 2 mL of saline was used to confirm the needle insertion, 20 mL of 0.25% bupivacaine was injected. The procedure was duplicated on the contralateral side.

#### 2.4.2. ESP Group

The pre-block preparations conducted for the M-TLIP group were similarly executed for patients undergoing the ESP block. The convex ultrasound probe was oriented sagittally to locate the spinous process at the level designated for discectomy. The probe was subsequently advanced 3–5 cm laterally to visualize the transverse processes of the target vertebra. An in-plane technique was employed to advance a 20 G 80–100 mm block needle in a craniocaudal direction until it made contact with the transverse process. To verify proper placement through hydrodissection, 2 mL of saline was utilized. Thereafter, 20 milliliters of bupivacaine (0.25% solution) were given (Figure 1). The procedure was duplicated on the contralateral side.

The total amount of bupivacaine administered bilaterally in all patients (100 mg) remained well below the recommended maximum safe limit of 2.5 mg/kg, ensuring that no participant was exposed to potentially toxic doses.

### 2.5. Surgical Technique

Using the standard microdiscectomy procedure, the same surgical team operated on each patient.

### 2.6. Anesthesia Induction and Maintenance

General anesthesia induction involved intravenous administration of midazolam (0.1 mg/kg), propofol (2–3 mg/kg), fentanyl (100 μg), and rocuronium (0.6 mg/kg). No additional fentanyl was routinely administered after induction; however, intraoperative hemodynamic responses were treated as needed. Specifically, an increase in mean blood pressure of ≥20% from baseline was considered indicative of inadequate analgesia and was treated with a rescue dose of fentanyl (1 μg/kg). This practice ensured that the total fentanyl dose for each patient remained below the recommended safety threshold of 2 μg/kg whenever rescue administration was required. During the study period, no patient required rescue fentanyl administration, and intraoperative opioid exposure therefore remained standardized across groups. After achieving adequate neuromuscular relaxation, patients were intubated with an appropriately sized endotracheal tube. Maintenance of anesthesia was achieved with sevoflurane at 1 MAC in a 50% oxygen–air mixture with a fresh gas flow of 3 L/min. All patients received 1000 mg intravenous paracetamol and 8 mg ondansetron during closure.

### 2.7. Postoperative Period

We noted the durations of the procedure and anesthesia for all patients and extubated them once the surgery was complete. Moving on to the post-anesthesia care unit (PACU) was the next step for the patients.

Postoperatively, patients were routinely administered 1000 mg intravenous paracetamol every 8 h. If VAS ≥ 4 one hour after paracetamol administration, 100 mg intravenous tramadol was administered; patients who did not achieve adequate relief within 30 min received an additional 100 mg dose. Total tramadol consumption within the first 24 h was recorded. VAS was used to quantify postoperative pain intensity. The scale ranged from 0 (no pain) to 10 (worst imaginable pain) [6]. Pain scores measured at 1, 2, 4, 8, and 24 h were defined as the primary outcome.

The patients’ sedation–agitation levels from extubation to arrival in the PACU were assessed and documented with sedation–agitation status graded on the Riker Sedation–Agitation Scale (RSAS), where 1 represents an unarousable state and 5 denotes dangerous agitation [7]. The patients were simultaneously assessed for postoperative nausea and vomiting (PONV) using a 2-point scale (0 = none, 1 = present) alongside VAS scores. Those with a positive PONV score were treated with a 4 mg intravenous dose of ondansetron. The amount of tramadol administered within the initial 24 h following surgery was calculated, and patient satisfaction was measured using a 5-point Likert scale ranging from 1 (extremely dissatisfied) to 5 (very satisfied) [8]. These data were recorded as secondary outcomes along with intraoperative fentanyl consumption.

### 2.8. Sample Size Calculation

Sample size estimation was conducted using G*Power software (version 3.1.9.4, Kiel University, Kiel, Germany) [9]. The calculation was based on a two-tailed α level of 0.05 and a statistical power of 80%. As no prior head-to-head comparison between M-TLIP and ESP blocks in lumbar discectomy was available at the time of study design, the effect size (0.6788) was derived from previously reported 24 h VAS values in a randomized study comparing modified and classical TLIP techniques (mean ± SD: 0.37 ± 0.4 vs. 0.13 ± 0.3) [10]. The 24 h time point was selected because it represents a clinically relevant and commonly reported postoperative outcome measure in prior TLIP literature. Assuming an equal allocation ratio (N2/N1 = 1), a minimum total sample size of 72 participants was required. Although the primary outcome included repeated postoperative VAS measurements, basing the calculation on a single conservative time point was considered methodologically appropriate. Moreover, repeated-measures designs generally provide greater statistical efficiency, potentially increasing the effective power of the study.

### 2.9. Statistical Analyses

SPSS 16.0 for Windows (SPSS Inc., Chicago, IL, USA) was used for the statistical analysis. Kolmogorov–Smirnov test was used to determine whether the numerical data were normally distributed. Continuous data are expressed as mean and standard deviation; categorical variables were expressed as frequencies and percentages. Non-normally distributed variables are reported as median with interquartile range (IQR). Categorical data were expressed as case numbers or percentages (%) and analyzed using the χ^2^ test. For comparisons between groups, Student’s *t*-test was applied to normally distributed data, while the Mann–Whitney U test was used for non-normally distributed data. In all comparisons, a *p*-value < 0.05 was considered statistically significant. Tramadol consumption was not normally distributed and was therefore analyzed using the Mann–Whitney U test. Repeated postoperative VAS measurements were analyzed using a linear mixed-effects model to account for within-subject correlations over time. Time was treated as a categorical fixed effect, group (M-TLIP vs. ESP) as a fixed effect, and subject as a random intercept. Interaction between group and time was also examined. Estimated marginal means with 95% confidence intervals were reported. A two-sided *p*-value < 0.05 was considered statistically significant.

Baseline demographic and clinical characteristics were compared between groups to assess potential imbalance. Given the absence of significant differences in measured baseline variables, additional multivariable adjustment was not performed.

## 3. Results

**Figure 2:**
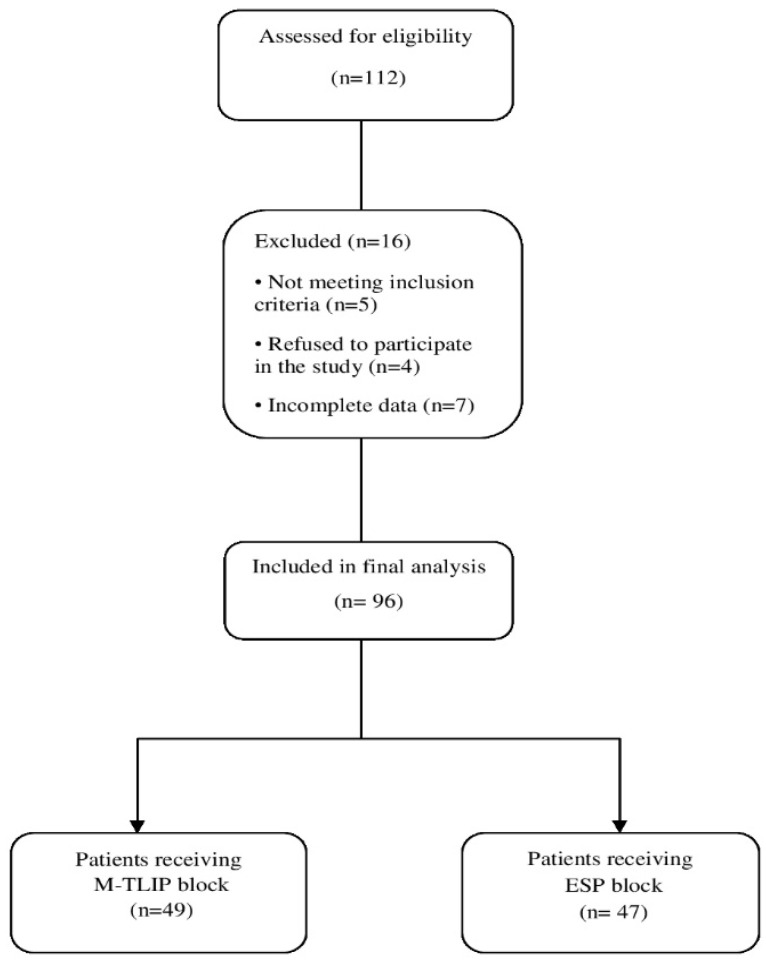
Flow diagram of participant inclusion and analysis according to STROBE recommendations.

A total of 112 patients, aged 18 to 70 years with ASA physical status I–III, were screened for eligibility to undergo lumbar discectomy. Nine patients were excluded prior to enrollment due to refusal to participate or failure to meet inclusion criteria, including infection at the application site, coagulopathy, morbid obesity (body mass index > 35 kg/m^2^), ASA classification greater than III, allergy to local anesthetics, chronic pain, inability to provide written consent, long-term opioid use, psychiatric history, or requirement for emergency surgery. During follow-up, four patients (one in the M-TLIP group and three in the ESP group) were excluded due to incomplete data collection. Consequently, 96 patients were included in the final analysis (Figure 2).

Of the analyzed cohort, 60 patients were female and 36 were male, with a mean age of 42.13 ± 11.55 years. The mean duration of surgery and anesthesia were 68.57 ± 7.35 and 86.94 ± 10.91 min, respectively. Age, sex, BMI, and the durations of surgery and anesthesia were comparable between groups, with no statistically significant differences observed (*p* = 0.99, 0.99, 0.37, 0.16, 0.13 respectively) (Table 1).

There was also no statistically significant difference in postoperative pain scores or opioid consumption between patients undergoing one-level and two-level laminectomy; therefore, both were analyzed together as predefined in the study protocol.

**Figure 3:**
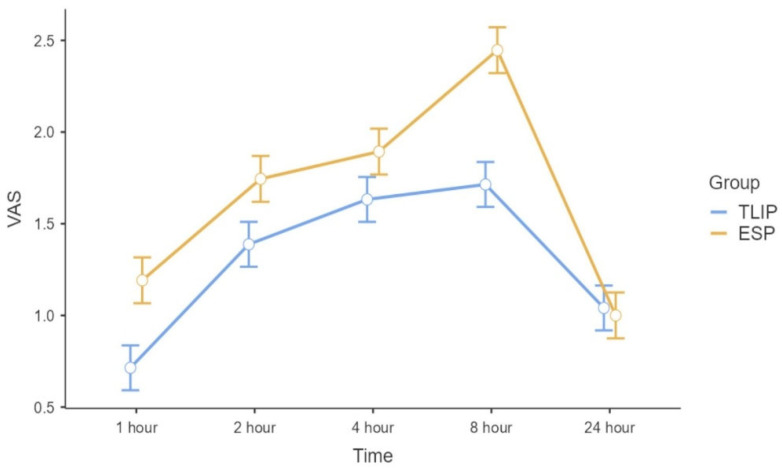
Estimated marginal mean VAS scores over postoperative time in the M-TLIP and ESP groups. Pain scores were assessed at postoperative 1, 2, 4, 8, and 24 h. Error bars represent 95% confidence intervals. VAS: Visual Analog Scale (0–10).

Postoperative pain scores were analyzed using a linear mixed-effects model to account for repeated measurements within subjects. The analysis revealed a significant main effect of group, with the ESP group demonstrating higher VAS scores compared with the M-TLIP group (estimate = 0.36, 95% CI 0.17–0.54, *p* < 0.001). A significant main effect of time was observed, with VAS scores increasing at 2, 4, and 8 h postoperatively compared with 1 h (all *p* < 0.001), while no significant difference was observed at 24 h (*p* = 0.56). The group × time interaction was significant only at 24 h (*p* = 0.027), indicating that the difference in VAS scores between groups diminished at this time point (Table 2; Figure 3). Estimated marginal means of VAS scores over time for each group are presented in Table 3).

Intraoperative fentanyl consumption was identical across groups, as no patient required rescue fentanyl administration during surgery (100 μg in all patients).

Postoperative tramadol use differed between groups. Only 2 of 49 patients (4.1%) in the M-TLIP group required tramadol within the first 24 h compared with 8 of 47 patients (17.0%) in the ESP group (*p* = 0.048, Fisher’s exact test). Consistent with this finding, total tramadol consumption demonstrated a significant between-group difference when analyzed using the Mann–Whitney U test (*p* = 0.039) (Table 4).

Postoperative agitation levels were similar, with both groups awakening in a cooperative and calm state (*p* = 0.82) (Table 5). Patient satisfaction scores also showed no significant difference (*p* = 0.53) (Table 5).

Blood pressure and heart rate values remained statistically similar between groups during both intraoperative and postoperative periods (*p* > 0.05). Nausea and vomiting were observed in 2 of 49 patients who underwent M-TLIP, but not in patients who underwent ESP (*p* = 0.30) (Table 6).

## 4. Discussion

The present study demonstrated that postoperative pain intensity, particularly during the early postoperative period and 24 h opioid consumption were significantly lower in patients receiving the M-TLIP block compared with those treated with the ESP technique. The lower proportion of patients requiring tramadol in the M-TLIP group further suggests a reduced need for rescue opioid analgesia during the early postoperative period. When pain trajectories were evaluated using a linear mixed-effects model, the analgesic advantage of M-TLIP was most pronounced in the early postoperative hours, while the between-group difference diminished by the 24th postoperative hour. In contrast, sedation–agitation levels and patient satisfaction scores were comparable between groups. These findings suggest that, even in the absence of randomization, M-TLIP may offer a clinically meaningful analgesic advantage over ESP block in routine lumbar discectomy practice. Importantly, this comparison reflects real-world clinical conditions rather than protocol-driven settings, thereby enhancing the external validity of the findings.

With the expanding use of ultrasound guidance in anesthetic practice, interfascial plane blocks have gained increasing attention as components of multimodal analgesia for spine surgery. However, the number of plane blocks applicable to lumbar discectomy remains limited, with ESP, TLIP, and M-TLIP being the most commonly utilized techniques.

Previous studies evaluating ESP block in lumbar discectomy have reported variable results. While some investigations demonstrated reductions in postoperative pain scores and opioid consumption, others reported modest or inconsistent analgesic benefits. A recent meta-analysis further highlighted the heterogeneity of ESP block outcomes in lumbar spine surgery, underscoring the need for additional comparative investigations [11,12,13,14]. These inconsistent findings suggest that the analgesic efficacy of ESP block may be influenced by surgical technique, tissue disruption, and postoperative anatomical changes.

Since its initial description, the TLIP block and its modified version have been investigated as alternatives for postoperative analgesia in lumbar spine surgery. Early volunteer and randomized studies demonstrated effective sensory blockade and reduced postoperative opioid requirements with TLIP and M-TLIP techniques [4,15]. Subsequent studies confirmed that the modified approach provides effective analgesia while potentially reducing the risk of neuraxial injury [16,17]. In contrast to these primarily randomized or controlled settings, the present study contributes prospective observational comparative data obtained under routine clinical conditions, thereby complementing the existing literature.

Concerns regarding ESP block have also been raised. It has been suggested that local anesthetic spread during ESP block may affect both dorsal and ventral rami, potentially leading to unintended motor blockade. In addition, surgical dissection of the erector spinae muscles during lumbar discectomy may facilitate anesthetic leakage from the injection plane, resulting in reduced block efficacy or unpredictable spread [18,19]. Neurophysiological studies have further supported the safety profile of the modified TLIP technique by demonstrating preserved SSEP and MEP signals, whereas alterations have been observed following classical TLIP application [20].

Although most studies on TLIP and M-TLIP blocks have focused on single-level lumbar procedures, their applicability in multilevel surgery remains an area of interest. Experimental and clinical evidence has demonstrated cranio-caudal spread over multiple vertebral levels following M-TLIP injection, with effective sensory blockade lasting up to 24 h [5,21,22,23]. In the present series, no significant differences in postoperative pain scores or opioid consumption were observed between single-level and two-level procedures, supporting the decision to analyze all patients as a single cohort, as predefined in the study protocol.

Although the reductions in VAS scores and tramadol consumption observed with M-TLIP reached statistical significance, the magnitude of these differences was modest. The largest between-group difference in VAS scores was observed at 8 h postoperatively (0.73 points on a 10-point scale), while differences at earlier time points ranged between 0.26 and 0.48 points. According to the systematic review by Laigaard et al. [24], the clinician-perceived MCID for acute postoperative pain commonly ranges between 15–18 mm on a 100 mm scale, corresponding to approximately 1.5–1.8 points on a 10-point scale. Therefore, the observed differences did not exceed commonly reported MCID thresholds. Nevertheless, the consistent reduction in early postoperative pain scores, together with the lower proportion of patients requiring rescue tramadol, may still be clinically relevant within a multimodal analgesic framework.

From a mechanistic perspective, although the anatomical spread of injectate may vary, the M-TLIP technique may facilitate retention of local anesthetic within a compartment farther from the surgical field, potentially reducing leakage and contributing to prolonged analgesic effects. In contrast, postoperative anatomical alterations following laminectomy may disrupt the posterior fascial planes, limiting predictable cephalocaudal spread of local anesthetic in the ESP plane. Recent clinical evidence appears to be consistent with this hypothesis, reporting reduced or inconsistent ESP block performance in post-laminectomy patients [25,26]. Collectively, these observations suggest that surgical anatomy may play an important role in determining the relative analgesic effectiveness of interfascial plane blocks in lumbar discectomy. These anatomical considerations may partly explain why the analgesic advantage of M-TLIP was more evident in the early postoperative phase, whereas the difference between techniques became less pronounced at later time points.

## 5. Study Limitations

This study has several limitations. First, the single-center design and relatively modest sample size may limit the generalizability of the findings. Second, the absence of randomization and formal double-blinding, inherent to the observational study design, restricts causal inference and may allow residual confounding. Because postoperative pain assessment relied on subjective VAS scoring, the absence of formal blinding may have introduced measurement bias. Although block allocation was not randomized, all procedures were performed by a single experienced anesthesiologist, which reduced inter-operator variability and enhanced procedural consistency. Furthermore, the lack of intraoperative somatosensory and motor evoked potential (SSEP and MEP) monitoring precluded objective assessment of potential interactions between sensory and motor pathways. Future prospective, multicenter randomized controlled trials with larger sample sizes are warranted to further clarify the comparative efficacy of M-TLIP and ESP blocks, particularly in two-level and multilevel lumbar spine surgeries. Additionally, the sample size calculation was based on effect sizes derived from a prior TLIP comparison rather than a direct ESP comparison, which may affect precision of the estimated effect size. Although baseline characteristics were comparable between groups, the absence of multivariable adjustment may allow residual confounding inherent to observational study designs.

## 6. Conclusions

In this prospective observational comparative study, M-TLIP block was associated with lower early postoperative pain scores during the early postoperative period and reduced 24 h opioid consumption compared with ESP block in patients undergoing lumbar discectomy. The analgesic advantage of M-TLIP was most evident in the early postoperative phase, with diminishing differences between techniques by 24 h. These findings suggest that M-TLIP may represent a clinically useful component of multimodal analgesia in routine lumbar discectomy practice. Further multicenter randomized controlled studies are warranted to confirm these findings and to define the optimal role of M-TLIP in multilevel lumbar spine surgery. These findings may have practical implications for anesthesiologists seeking opioid-sparing regional analgesic strategies in lumbar spine surgery.

## Figures and Tables

**Table 1:** Comparison of patients according to demographic and intraoperative characteristics.

	Group M-TLIP (*n* = 49)	Group ESP (*n* = 47)	*p*
Ages (years)	42.12 ± 10.32	42.14 ± 12.82	0.99
Body mass index (kg/m^2^)	26.08 ± 3.35	25.47 ± 3.40	0.37
Surgery duration (min)	69.85 ± 8.21	67.23 ± 6.14	0.16
Anesthesia duration (min)	85.41 ± 9.38	88.38 ± 10.71	0.13
Gender (*n*/%)			0.99
Female	31 (32.3)	29 (30.2)	
Male	18 (18.8)	18 (18.8)	
Smoking (*n*/%)			0.4
Yes	25 (26)	20 (20.8)	
No	24 (25)	27 (28.1)	
Comorbidity (*n*/%)			0.91
Yes	10 (10.4)	10 (10.4)	
No	39 (40.6)	37 (38.5)	
ASA (*n*/%)			0.42
I	17 (17.7)	20 (20.8)	
II	32 (33.2)	27 (28.1)	
Number of discectomy levels			0.68
Single level (*n*/%)	46 (47.9)	45 (46.87)	
Two level (*n*/%)	3 (3.12)	2 (2.08)	

Data are presented as mean ± standard deviation (SD) or number (percentage). M-TLIP: Modified thoracolumbar interfascial plane block, ESP: Erector spinae plane block, ASA: American Society of Anesthesiologists risk classification.

**Table 2:** Fixed effects from the linear mixed-effects model for postoperative VAS scores.

	Estimate	SE	95% CI	df	*p*
Group (ESP vs. TLIP)	0.36	0.09	0.17–0.54	94	<0.001
Time (2 h vs. 1 h)	0.61	0.12	0.38–0.84	376	<0.001
Time (4 h vs. 1 h)	0.81	0.12	0.58–1.04	376	<0.001
Time (8 h vs. 1 h)	1.13	0.12	0.90–1.36	376	<0.001
Time (24 h vs. 1 h)	0.07	0.12	−0.16–0.30	376	0.56
Group × Time (24 h)	−0.52	0.23	−0.98–−0.06	376	0.027

Values represent fixed effects derived from a linear mixed-effects model with random intercepts for subjects to account for repeated measurements. Time was treated as a categorical variable with postoperative 1 h as the reference category.

**Table 3:** Estimated marginal means of postoperative VAS scores by group and time derived from the linear mixed-effects model.

Time	Group M-TLIP (Mean ± SE)	Group ESP (Mean ± SE)
1 h	0.71 ± 0.06	1.19 ± 0.12
2 h	1.39 ± 0.11	1.74 ± 0.13
4 h	1.63 ± 0.12	1.89 ± 0.14
8 h	1.71 ± 0.13	2.44 ± 0.16
24 h	1.04 ± 0.09	1.00 ± 0.11

Estimated marginal means ± standard errors obtained from the linear mixed-effects model adjusting for time and group effects, with subject included as a random effect to account for repeated measurements.

**Table 4:** Comparison of intraoperative and postoperative opioid use of patients.

	Group M-TLIP (*n* = 49)	Group ESP (*n* = 47)	*p*
Intraoperative fentanyl consumption (μg)	100 (identical in all patients)	100 (identical in all patients)	-
Postoperative tramadol consumption (mg), median (IQR)	0 (0-0)	0 (0-0)	0.0397 *
Patients requiring tramadol, *n* (%)	2 (4.1%)	8 (17.0%)	**0.048 ^†^**

Data are presented as median (interquartile range) or number (percentage), as appropriate. * Postoperative tramadol consumption was compared using the Mann–Whitney U test due to its highly skewed distribution. ^†^ The proportion of patients requiring tramadol was compared using Fisher’s exact test. Bold values indicate statistical significance (*p* < 0.05). Intraoperative fentanyl consumption was identical across groups (100 μg in all patients), as no rescue fentanyl was required. M-TLIP: Modified thoracolumbar interfascial plane block, ESP: Erector spinae plane block.

**Table 5:** Comparison of groups according to sedation agitation and patient satisfaction score.

	Group M-TLIP (*n* = 49)	Group ESP (*n* = 47)	*p*
**RSAS score (*n*/%)**			0.82
Unarousable	1 (2.0%)	1 (2.1%)	
Sedated	14 (28.6%)	16 (34.0%)	
Calm and Cooperative	32 (65.3%)	27 (57.4%)	
Agitated	2 (4.1%)	2 (4.3%)	
Dangerous Agitation	0 (0)	1 (2.1%)	
**Patient satisfaction score (*n*/%)**	5 (10.2%)	5 (10.6%)	0.53
Very satisfied	28 (57.1%)	22 (46.8%)	
Satisfied	15 (30.6%)	19 (40.4%)	
Neither satisfied/dissatisfied	1 (2%)	0 (0)	
Dissatisfied	0 (0)	1 (2.1%)	

Data are presented as number and percentage (%). Chi-square test was used to compare RSAS and patient satisfaction score data between groups and to determine the results in percentage (n%). RSAS: Riker’s Agitation Sedation Scale, M-TLIP: Modified thoracolumbar interfascial plane block, ESP: Erector spinae plane block.

**Table 6:** Comparison of postoperative nausea and vomiting between groups.

	Group M-TLIP (*n* = 49)	Group ESP (*n*= 47)	*p*
Side effect-PONV (*n*/%)			0.30
none	47 (95.9%)	47 (100%)	
yes	2 (4.1%)	0 (0)	

Data are presented as number and percentage. Chi-square test was used to compare PONV data between groups and to determine the results in percentage (*n*/%). M-TLIP: Modified thoracolumbar interfascial plane block, ESP: Erector spinae plane block, PONV: postoperative nausea and vomiting.

## Data Availability

Data available on request from the authors.

## Supplementary Materials

The following supporting information can be downloaded at: https://www.mdpi.com/article/10.3390/jcm15062214/s1, File S1: STROBE Checklist.
